# Non-pharmaceutical interventions in containing COVID-19 pandemic after the roll-out of coronavirus vaccines: a systematic review

**DOI:** 10.1186/s12889-024-18980-2

**Published:** 2024-06-06

**Authors:** Xiaona He, Huiting Chen, Xinyu Zhu, Wei Gao

**Affiliations:** 1 Department of Epidemiology and Health Statistics, School of Public Health, Jiangxi Medical College, Nanchang University, Nanchang, China; 2 Jiangxi Provincial Key Laboratory of Preventive Medicine and Public Health, Nanchang University, No. 461, Bayi Ave,, Nanchang, 330006 PR China

**Keywords:** Optimal strategies, Non-pharmaceutical interventions, Vaccination, Real-world impact, COVID-19

## Abstract

**Background:**

Non-pharmaceutical interventions (NPIs) have been widely utilised to control the COVID-19 pandemic. However, it is unclear what the optimal strategies are for implementing NPIs in the context of coronavirus vaccines. This study aims to systematically identify, describe, and evaluate existing ecological studies on the real-world impact of NPIs in containing COVID-19 pandemic following the roll-out of coronavirus vaccines.

**Methods:**

We conducted a comprehensive search of relevant studies from January 1, 2021, to June 4, 2023 in PubMed, Embase, Web of science and MedRxiv. Two authors independently assessed the eligibility of the studies and extracted the data. A risk of bias assessment tool, derived from a bibliometric review of ecological studies, was applied to evaluate the study design, statistical methodology, and the quality of reporting. Data were collected, synthesised and analysed using qualitative and quantitative methods. The results were presented using summary tables and figures, including information on the target countries and regions of the studies, types of NPIs, and the quality of evidence.

**Results:**

The review included a total of 17 studies that examined the real-world impact of NPIs in containing the COVID-19 pandemic after the vaccine roll-out. These studies used five composite indicators that combined multiple NPIs, and examined 14 individual NPIs. The studies had an average quality assessment score of 13 (range: 10–16), indicating moderately high quality. NPIs had a larger impact than vaccination in mitigating the spread of COVID-19 during the early stage of the vaccination implementation and in the context of the Omicron variant. Testing policies, workplace closures, and restrictions on gatherings were the most effective NPIs in containing the COVID-19 pandemic, following the roll-out of vaccines. The impact of NPIs varied across different time frames, countries and regions.

**Conclusion:**

NPIs had a larger contribution to the control of the pandemic as compared to vaccination during the early stage of vaccine implementation and in the context of the omicron variant. The impact of NPIs in containing the COVID-19 pandemic exhibited variability in diverse contexts. Policy- and decision-makers need to focus on the impact of different NPIs in diverse contexts. Further research is needed to understand the policy mechanisms and address potential future challenges.

**Supplementary Information:**

The online version contains supplementary material available at 10.1186/s12889-024-18980-2.

## Background

Since the availability of COVID-19 vaccines, governments worldwide have implemented vaccination and non-pharmaceutical interventions (NPIs) such as testing policies, gathering restrictions, facial covering policies, school closures, workplace closures to contain local transmission of COVID-19 [1, 2]. The NPIs, also known as public health measures, aim to break infection chains by altering key aspects of our behavior. Extensive research has been dedicated to examining the impact of NPIs in controlling the outbreak of COVID-19 [3–5].

Before the COVID-19 pandemic, there existed literature in addressing the impact of NPI implementation on influenza pandemic [6]. However, a key challenge in this topic is the limited evidence regarding the impact of NPIs, which predominantly relies on mathematical modelling with a limited number of empirical studies [7–9].

Considering the potential harm posed by respiratory infectious disease outbreaks and the high social and economic costs associated with implementing various NPIs, it is essential to conduct research that examines the impact of NPIs in controlling pandemics in real-world settings. Mendez et al. conducted a systematic review and identified that school closures, workplace closures, business and venue shutdowns, and public event restrictions as the most effective measures in controlling the real-world spread of COVID-19 [7].

However, various countries implemented diverse NPIs at different stages of the pandemic to control the spread of COVID-19, especially after the introduction of coronavirus vaccines. Asian countries consistently enforced strict NPIs throughout the first half of 2021 [10], while no NPIs were implemented in France after May 2021 [11]. At the early stage of vaccine roll-out, vaccination coverage in most countries remained relatively low [2]. As of June 30, 2021, a total of 29.29% of the world’s population had received at least one dose of the vaccine, with significant variations in vaccination coverage across countries [2]. Despite an increase in vaccination rates in many countries during the latter half of 2021, the number of confirmed new COVID-19 cases remained high worldwide due to the prevalence of the highly transmissible and immune-escape Delta variant in the second half of 2021 [12], followed by the emergence of the Omicron variant in early 2022 [13]. Yet, with the increase in COVID-19 vaccination rates, there has been a reduction in mortality and morbidity despite the high level of transmission. This indicates that widespread vaccine coverage has played a positive role in mitigating the health impacts of the disease.

The impact of NPIs in controlling the COVID-19 pandemic after the roll-out of vaccines has also received considerable attention [14]. Nevertheless, the policy mechanisms underlying their impact, such as determining when to implement stricter lockdown measures or when to ease restrictions, as well as identifying which types of NPIs are more suitable for different stages, remain unclear.

This review focuses on investigating the real-world impact of NPIs in containing the COVID-19 pandemic after vaccine roll-out, in order to search for optimal strategies for implementing NPIs. We summarize the current evidence from the real world on the impact, aiming to deepen the current understanding, fill in the gaps in the topics, and provide evidence for the future.

## Methods

The reporting of this review was guided by the Preferred Reporting Items for Systematic Reviews and Meta-Analyses (PRISMA) statement [15]. See Supplemental file for further details. This review was registered at the international prospective register of systematic reviews (PROSPERO; CRD42023411560).

### Data sources and searches

We conducted a comprehensive search of relevant literature in Embase, PubMed, and Web of Science, and preprints on MedRxiv from January 1, 2021 to June 4 2023. Our search was limited to articles written in English. The search terms included NPIs, COVID-19 and vaccination, which were detailed in Table S1 of the supplemental file. We used EndNote (version 20.0) software to process and remove duplicates. In addition, we manually searched for citations and related articles of the included studies using Google Scholar.

### Study selection and eligibility criteria

One author (XH) screened eligible studies by reviewing the titles and/or abstracts of searched articles using EndNote (version 20.0). If an article was deemed relevant or if the information provided in the title or abstract was insufficient to make a decision, the full texts were retrieved and examined. For all eligible studies(*n* = 182), two independent authors (XH and HC) assessed the eligibility criteria for each study by evaluating the full text and determining inclusion or exclusion. Any discrepancies between authors were resolved by discussions with the third reviewer (XZ) and the senior author (WG) to reach a consensus.

In general, we adopted an inclusive approach by retaining all studies that could not be excluded with high confidence. All decisions were documented in a spreadsheet. Studies were included in the review if they: (1) assessed the impact of NPIs during the roll-out of COVID-19 vaccines; (2) evaluated the impact of NPIs and vaccination coverage using real-world data; (3) analyzed the respective/interactive impact of NPIs and vaccination coverage; (4) assessed the impact at least one type of NPIs; (5) measured at least one health outcome; (6) obtained evidence through ecological study. Studies were excluded from the review if they: (1) were based on forecasts or simulations; (2) analyzed the impact of adherence or compliance to NPIs and intention or willingness to vaccination; (3) assessed NPI impact in controlling other diseases; (4) did not directly assess the impact of NPIs.

### Quality assessment

To assess the quality of studies, we used a risk of bias assessment tool based on a bibliometric review of ecological studies, as proposed by Dufault et al. [16]. This tool has been previously used and adapted in recent reviews [7, 17, 18]. The purpose of the risk of bias assessment tool is to critically evaluate study design, statistical methodology and practices, and the quality of reporting. Two independent reviewers (XH and HC) evaluated the risk of bias for each included study. Any discrepancies between the reviewers were resolved by discussion with a third reviewer (XZ) and the senior author (WG) to reach a consensus. The checklist of risk of bias assessment tool was included in the Table S2 of Supplemental File.

### Data synthesis and analysis

Characteristics and outcomes of individual studies were extracted, including study authors, year, setting, study design, duration of study, type and/or intensity of NPIs, vaccination coverage, assessment indicators of outcome such as time varying reproduction number (Rt), the number of daily new cases or deaths. The classification and intensity of NPIs were mainly based on information from a global panel database of pandemic policies (Oxford COVID-19 Government Response Tracker) [1].

In the final stage of the systematic review, we synthesised the findings from all eligible ecological studies(*n* = 17) to determine the real-world impact of NPIs in containing the COVID-19 pandemic after the vaccine roll-out. Data were collected, synthesized, and analyzed using quantitative and qualitative approaches. Specifically, we collected the impact of NPIs and vaccination reported in each included study, and then summarized and compiled the main results. We have considered not only the impact of individual NPIs but also collected data on the impact measured by a composite indicator of NPIs. This holistic approach is supported by the fact that most countries implemented multiple NPIs as a package to mitigate the spread of COVID-19 during the pandemic. The results were presented using summary tables and figures, including the target countries and regions of the studies, NPIs types, evidence quality.

## Results

### Summary of literature screening and background

**Fig. 1:**
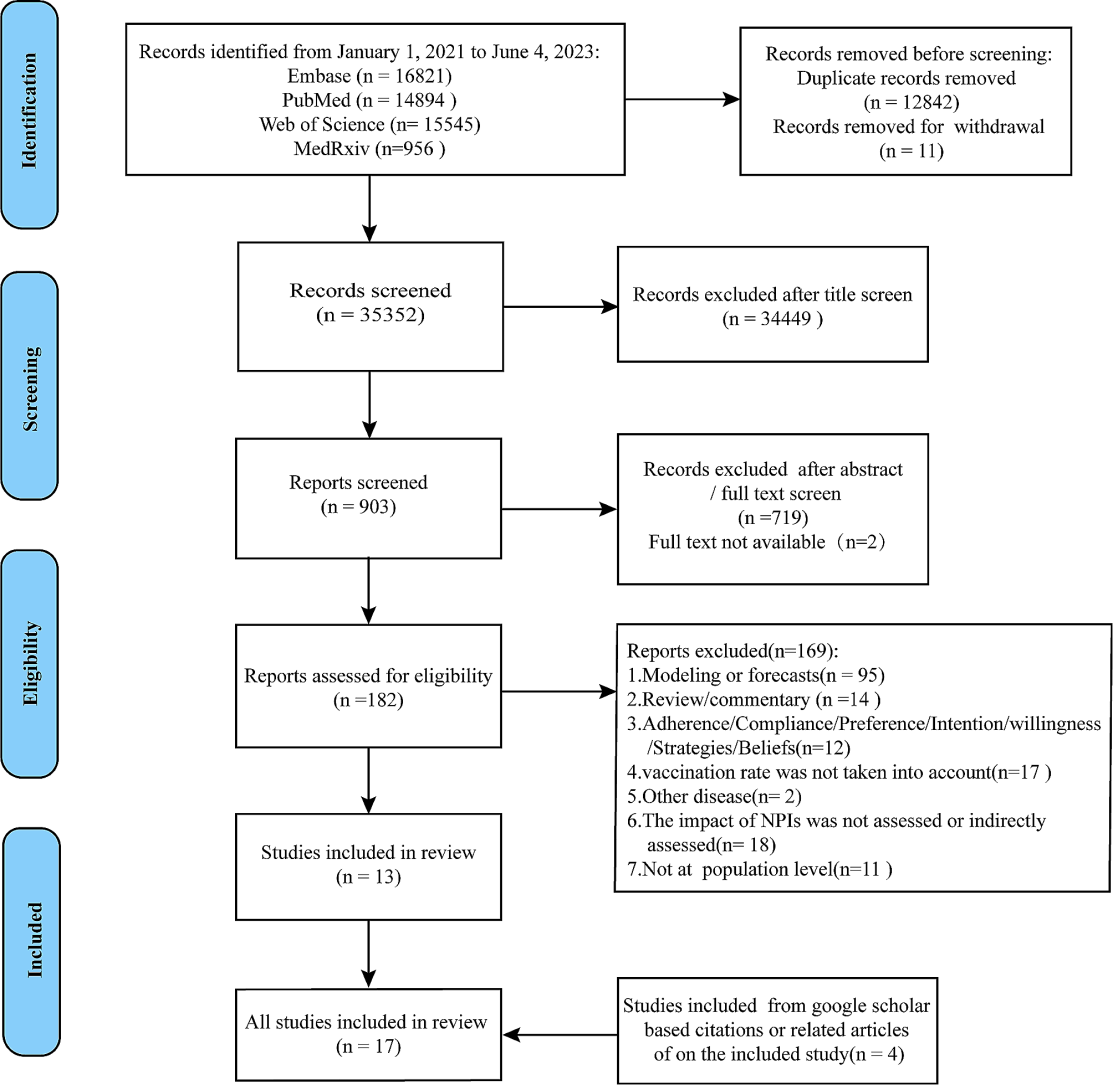
PRISMA flow diagram for the selection of studies

Seventeen ecological studies were included in the review, of which fourteen were published and three were preprints. The PRISMA diagram flow is presented in Fig. 1. For more information on excluded articles and reasons for their exclusion, please refer to Table S3 in the Supplemental File. These studies encompass research samples from over 88% of countries and regions worldwide, with each study focusing on a different geographical scope. Table 1 provides a breakdown of the studies: eight evaluated the impact of NPIs in containing the COVID-19 pandemic on a global scale [19–26], three focused on Europe [27–29], two on the United States [30, 31], one on Asia [10], and one each on India [32], France [11] and Korea [33].

The seventeen studies examined the impact of NPIs on the COVID-19 pandemic during different periods. Eight studies evaluated their impact during the early stage of vaccine roll-out (before July 2021), five during the later stage (the second half of 2021), and the remaining four during the Omicron stage (contain 2022 period).

**Table 1:** Background of included studies

Geographical scope	Number of studies	Study time		
		Early stage (First half of 2021)	Later stage (Second half of 2021)	Omicron stage
Worldwide	8	3	1	4
Europe-wide	3	1	2	0
US	2	2	0	0
Asia-wide	1	1	0	0
India	1	0	1	0
France	1	1	0	0
South Korea	1	0	1	0
Total	17	8	5	4

In terms of quality assessment, the seventeen studies received a moderately high score, averaging 13 (range: 10–16) out of a maximum score of 17. This score reflects the strength of the evidence. The primary sources of risk of bias were the quality of reporting, validity of regression, control of covariates, and internal validity of the methodology. Detailed assessment records can be found in Table S4. Studies used different statistical methods and outcomes, and conducted sensitivity analyses. Only a few studies considered the impact of seasonality.

### Study characteristics

Researchers examined various types of NPIs in the seventeen identified studies. Nine studies evaluated the overall impact of a composite indicator of NPIs (Table 2), while twelve studies specifically assessed the impact of individual NPIs (Table 3). Bollyky et al., Paireau et al.,  Li et al., and Hongjian Wang et al. analyzed both the impact of the composite indicator of NPIs and individual NPIs. More detailed information including the data sources and the analytical methods used in the studies can be found in Supplemental File (Table S5).

**Table 2:** Characteristics of studies evaluating the overall impact of a composite indicator of NPIs

Author	Publication status, year	Geographical scope	NPIs	Outcome(s)	Score
Ge et al. (1)	Published, 2022	European, 31 countries	Stringency index^a^	Rt	16
Bollyky et al.	Published, 2023	US 50 states and Washington, DC	Policy mandates^b^	Cases, Deaths	16
Zhou et al.	Published, 2022	European, 22 countries	Stringency index	Cases, deaths, excess mortality	15
Paireau et al.	Published, 2023	France, 92 departments	Lockdown^c^	Rt	15
Caixia Wang et al.	Published, 2023	Worldwide, 176 countries/territories	Stringency index	Deaths	14
Li et al.	Published, 2022	Worldwide, 8 countries	Combination of four NPIs^d^	Rt	14
Hale et al.	Published, 2021	Worldwide, 10 countries	Stringency index	Deaths	13
Hongjian Wang et al.	Published, 2023	Worldwide, 176 countries/territories	Stringency index	Rt	11
Kijin Kim et al.	Published, 2023	Korea	Social distancing policy index^e^	Cases	10

Notes: a = stringency index included eight containment and closure, including school closure, workplace closure, cancel public events, restrictions on gathering size, close public transport, stay-at-home requirements, restrictions on internal movement, restrictions on international travel; b = a summary measure that captures a state’s use of physical distancing and mask mandates;c = a comprehensive measure captures the level of social distancing; d = the combination of school closure, workplace closure, restrictions on mass gatherings and stay-at-home requirements; e = restrictiveness of government containment and closure measures implemented; Rt = time varying reproduction number

**Table 3:** Characteristics of studies evaluating the impact of individual NPIs

Author	Publication status, year	Geographical scope	NPIs	Outcome(s)	Score
Bollyky et al.	Published, 2023	US 50 states and Washington, DC	Closures of bars, restaurants, gyms, and schools, facial covering and vaccine mandates, and stay-at-home orders and gathering restrictions	Cases, deaths	16
Huy et al.	Published, 2022	Asina, 28 countries	School closure, workplace closure, public event canceling, public transport closure, stay at home requirements, restrictions on internal movement, international travel controls, public information campaign indicators, testing policy, contact tracing, and facial covering	Growth rate	15
Ge et al. (2)	Preprint, 2022	Worldwide, 63 countries	School closures, workplace closures, gathering restrictions, movement restrictions, public transport closures, international travel restrictions, and facial coverings	Decay ratio	15
Ertem et al.	Published, 2023	US, 2954 counties	Facial covering policies	Cases	15
Paireau et al.	Published, 2023	France, 92 departments	Curfews, school closures	Rt	15
Liang et al.	Published, 2021	Worldwide, 137 countries/territories	School closures, workplace closures, cancellation of public events, restrictions on gathering size, requirements to stay-at-home, and restrictions on international travel, public information campaigns, testing policy, contact tracing, face covering	Case doubling time	14
Shin et al.	Preprint, 2023,	India	Testing (Testing ratio)	Cases	13
Li et al.	Published, 2022	Worldwide, 8 countries	School closure; workplace closure; restrictions on public events; restrictions on gatherings; closure of public transport; stay-at-home requirements; restrictions on internal movement; and international travel controls.	Rt	12
Hongjian Wang et al.	Published, 2023	Worldwide, 176 countries/territories	Testing	Rt	11
Sookhyun Kim et al.	Published, 2023	European, 35 countries	Facial covering policies	Cases	10
Nesteruk	Preprint, 2022	Worldwide, Japan, Ukraine, USA, Hong Kong China, mainland China, European and African countries	Testing (testing ratio)	Cases	10
Nesteruk et al.	Published, 2022	Worldwide, 44 European and 14 other countries and regions	Testing (testing ratio)	Cases	10

Notes: decay ratio = the decay ratio of COVID19 infections; case doubling time = the number of days required for the accumulated case number to double

The composite indicator of NPIs in this review primarily included five types: stringency index, policy mandates, social distancing policy index, lockdown, and combination of four NPIs (school closure, workplace closure, restrictions on mass gatherings, and stay-at-home requirements). The specific names for these measures varied depending on their sources. The data on NPIs in these studies mainly came from OxCGRT, with additional sources including The Yale State and Local COVID-19 restriction database, governmental websites, and others. These composite indicators were calculated by combining multiple containment and closure measures, representing the overall intensity of various containment and closure policies to some extent. The individual NPIs included containment and closure measures, as well as health systems indicators. Containment and closure measures primarily encompassed restrictions on gatherings, school closures, workplace closures, and stay-at-home requirements. The evaluated health systems indicators included testing policy, facial coverings, contact tracing, public information campaigns, and vaccine mandates.

The studies on vaccination included data on the administration of the first dose, full vaccination, and booster doses according to the vaccination protocol. Two studies did not explicitly specify the doses. The outcome assessed mainly included cases, deaths, Rt, and others.

### The impact of composite indicator of NPIs for containing the COVID-19 pandemic after the roll-out of COVID-19 vaccines

As shown in Table 4, even after the introduction of vaccines, the implementation of containment and closure measures continued to have a impact on curtailing the spread of COVID-19. Some studies also compared the impact of NPIs during that period with the impact of vaccination coverage in mitigating the transmission of COVID-19 within populations. Specifically, NPIs were found to have a larger impact than vaccination in mitigating the spread of COVID-19 during early stage of the vaccination implementation. In the latter half of 2021, the impact of NPIs had relatively diminished compared to the earlier stages. During the Omicron stage, the measures implemented to control the spread of COVID-19 had a larger impact than vaccination coverage. More detailed information is provided in Table S6.

In addition to assessing the impact of combinations of containment and closure measures in controlling the spread of COVID-19(cases and Rt), NPIs were also considered in reducing the number of COVID-19 related deaths. Bollyky et al. ‘s study did not find evidence supporting the impact of NPIs in reducing COVID-19 related deaths, while other studies (Zhou et al., Hale et al. and Caixia Wang et al.) suggested that NPIs could reduced the number of deaths.

**Table 4:** The impact of a composite indicator of NPIs in the studies

Stage	Author	Geographical scope	NPIs assessed	Outcome	Impact	Compare to vaccination coverage	Score
Early	Ge et al. (1)	European	Stringency index	Rt	reduction in Rt	larger	16
Early	Bollyky et al.	United States	Policy mandates	cases	reduction in cases	not compared	16
Early	Zhou et al.	European	Stringency index	cases	reduction in cases	larger	15
Early	Paireau et al.	France	Lockdown	Rt	reduction in Rt	larger	15
Early	Zhou et al.	European	Stringency index	deaths	reduction in deaths	larger	15
Early	Hale et al.	Worldwide	Stringency index	deaths	reduction in deaths	not compared	13
Early	Bollyky et al.	United States	Policy mandates	deaths	not impact	not compared	16
Later	Ge et al. (1)	European	Stringency index	Rt	reduction in Rt	smaller	16
Later	Li et al.	Worldwide	Combination of four NPIs	cases	reduction in cases	not compared	14
Later	Kijin Kim et al.	South Korea	Social distancing index	cases	reduction in cases	smaller	10
Omicron	Hongjian Wang et al.	Worldwide	Stringency index	Rt	reduction in Rt	larger	11
Omicron	Caixia Wang et al.	Worldwide	Stringency index	deaths	reduction in deaths	larger	14

Note: The impacts of NPIs are given by each article. For detailed information, see Table S6

### The relative impact of individual NPIs for containing the COVID-19 pandemic after the roll-out of COVID-19 vaccines

**Fig. 2:**
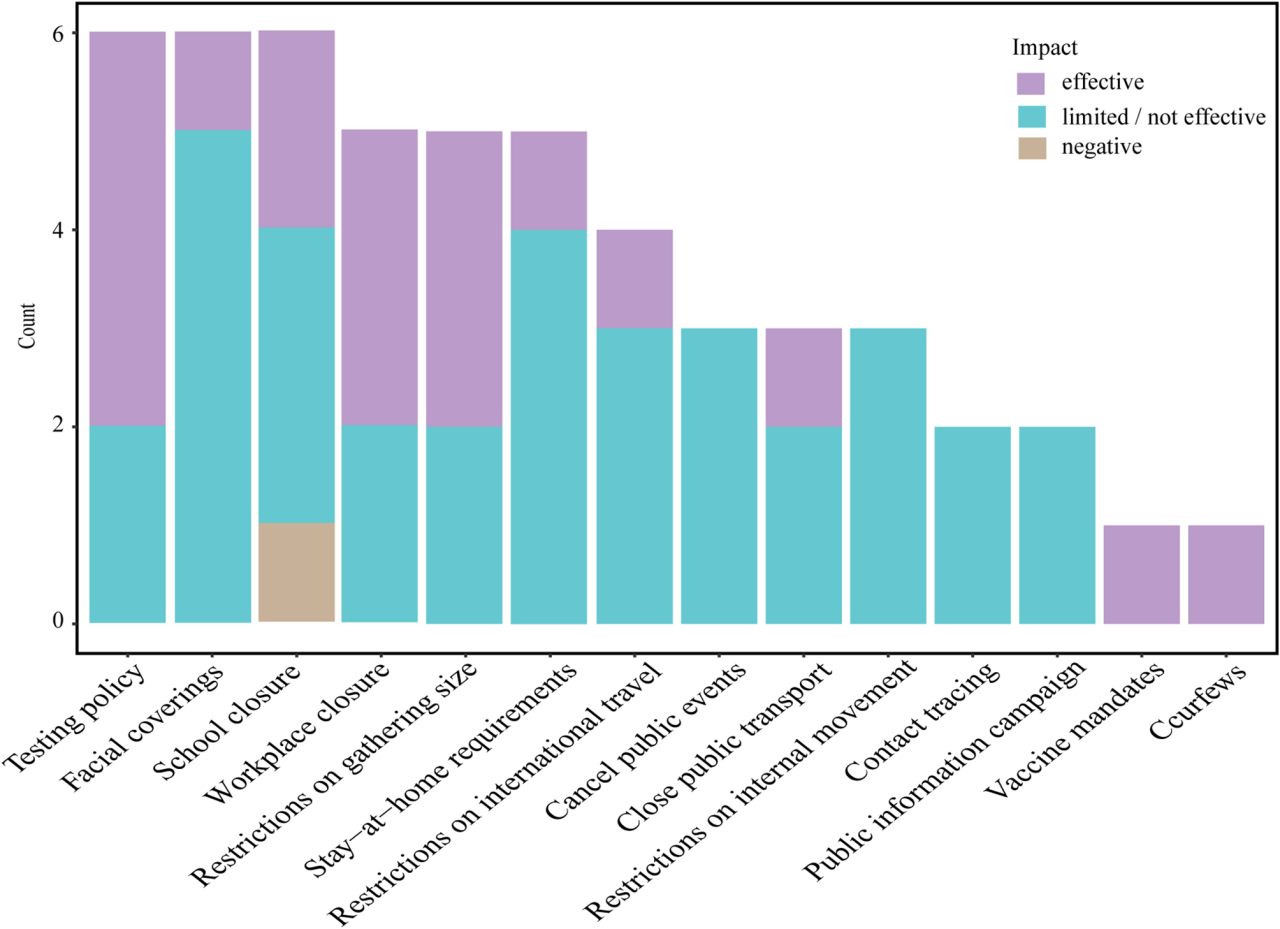
The relative impact of individual NPIs for containing the COVID-19 pandemic after the roll-out of COVID-19 vaccines. The Y-axis represents the count of assessments for this NPIs. The colors of the stacked bar represent the impact of assessed NPIs in containing the COVID-19 pandemic following the roll-out of vaccines. The purple color indicates that a study considers the NPI to be the most effective measure. The blue color signifies limited impact or a lack of association with containing the COVID-19 pandemic, according to the study. The brown color represents a negative correlation between the NPI and containing the spread of COVID-19

Twelve studies have evaluated the individual impacts of NPIs on controlling the COVID-19 pandemic after the roll-out of COVID-19 vaccines. A total of 14 individual NPIs were assessed in this review. Among them, testing policies, facial coverings, and school closures were mentioned most frequently. Additionally, workplace closures, restrictions on gatherings, stay-at-home requirements, and restrictions on international travel were also highlighted. Figure 2 demonstrates these findings.

Moreover, there is consistent evidence suggesting that testing policies (4/6), workplace closures (3/5), and restrictions on gatherings (3/5) may be the most effective containing the COVID-19 pandemic following the roll-out of vaccines.

We categorised the included studies according to the target countries and regions, as shown in Table 5. The types of NPIs evaluated and the impact NPIs identified varied across different geographical locations. Based on global studies, testing policy, restrictions on gathering size, workplace closure, school closure, stay-at-home requirements, and restrictions on international travel were found to be relatively effective. In Asian studies, restrictions on gathering size and the closure of public transport were considered as effective measures. In studies conducted in the United States, only vaccination mandates were deemed effective. Data from France indicated that curfews were effective. Face covering mandates were associated with a decrease in COVID-19 incidence in European countries. Testing was effective in India during the vaccination stage.

**Table 5:** The types and the relative impact individual NPIs were classified according to the study’s target countries and regions

Author	Geographical scope	NPIs assessed	Effective intervention measures	Score
Huy et al.	Asia	School closure, workplace closure, cancel public events, restrictions on gathering size, close public transport, stay-at-home requirements, restrictions on internal movement, restrictions on international travel, public information campaign, testing policy, contact tracing, facial coverings	Restrictions on gathering size, Close public transport	15
Liang et al.	Worldwide	School closure, workplace closure, cancel public events, restrictions on gathering size, stay-at-home requirements, restrictions on international travel , public information campaign, testing policy, contact tracing, facial coverings	School closure, workplace closure	14
Li et al.	Worldwide	School closure, workplace closure, cancel public events, restrictions on gathering size, close public transport, stay-at-home requirements, restrictions on internal movement, restrictions on international travel	School closure, workplace closure, restrictions on gathering size, stay-at-home requirements	12
Ge et al. (2)	Worldwide	School closure, workplace closure, restrictions on gathering size, close public transport, restrictions on international travel, facial coverings, restrictions on internal movement, stay-at-home requirements	Workplace closure, restrictions on gathering size, restrictions on international travel	15
Nesteruk	Worldwide	Testing policy	Testing policy	10
Nesteruk et al.	Worldwide	Testing policy	Testing policy	10
Hongjian Wang et al.	Worldwide	Testing policy	Testing policy	11
Bollyky et al.	US	Workplace closure, School closure, Restrictions on gathering size, Facial coverings, Stay-at-home requirements, Vaccine mandates, Restrictions on gathering size	Vaccine mandates	16
Ertem et al.	US	Facial coverings	None	15
Paireau et al.	France	Curfews, school closure	Curfews	15
Sookhyun Kim et al.	European	Facial coverings	Facial coverings	10
Shin et al.	India	Testing policy	Testing policy	13

## Discussion

### Summary of the main findings

The types of NPIs evaluated and the impact of NPIs identified varied across different periods and geographical locations. Overall, our research shows that NPIs continued to be effective in controlling the spread of COVID-19 even after the roll-out of vaccines. Our previous research work also supports this conclusion [34]. The most frequently evaluated NPIs included testing policies, facial coverings, and school closures, followed by workplace closures, restrictions on gatherings, stay-at-home requirements, and restrictions on international travel.

The overall impact of a composite indicator of NPIs varied depending on the period, with factors such as the intensity of implementation, compliance, increasing vaccine coverage, and the emergence of VOCs playing a role. NPIs remained important for mitigating the pandemic in the early stage of the vaccination when coverage was low [11, 27, 29]. However, as vaccine coverage increased, their marginal effects were surpassed by vaccination [27, 33]. In Omicron stage, measures were more effective in controlling the spread of COVID-19 than vaccination coverage due to the high immune evasion capability of the Omicron variant [13, 26]. It is important to note that NPIs and vaccinations work through different mechanisms to combat the pandemic [35]. NPIs physically reduce population contact and transmission of the virus, while vaccinations reduce susceptible populations by enhancing immunity. Overall, a combination of both containment and closure measures and vaccination is recommended to contain COVID-19 after the vaccine has been introduced [11, 21, 26, 27, 29, 33].

The types of the evaluated NPIs, as well as the effective NPIs, varied across different target countries and regions. Factors such as differences in government effectiveness [22], public awareness and behavioral responses to the prevention and control measures [21], and economic disparities among different countries and regions may affect the impact of various NPIs [36].

Testing policies is a central pillar of public health response to global health emergencies. In the included studies, testing policies were primarily evaluated during the Omicron period. Nesteruk [23], Nesteruk et al. [24], Wang et al. [26], and Shin et al. [32] found that strengthening testing could reduce the number of COVID-19 infections. Faster and decentralised nucleic acid testing technology may has the potential to be implemented on a larger scale in the community, help control the pandemic [37], reduce the need for strict control measures, and accelerate the recovery of social and economic activities. In addition, Shao et al. also found that large-scale SARS-CoV-2 rapid antigen testing alleviated the Omicron outbreak in China [38]. Moreover, it is crucial to ensure accessibility to COVID-19 tests (e.g., availability and familarity with COVID-19 tests), bolster public confidence in governmental control measures, and increase understanding of and perceived susceptibility to COVID-19 [39–42]. These efforts collectively help reduce barriers to testing, improve public willingness, and ultimately encourage individuals to participate in testing voluntarily.

Facial coverings are a form of personal protective equipment used to shield the face from various external hazards like splashes, droplets, and aerosols. Among the summarized evidence, only one study [28] found that the incidence of COVID-19 was significantly higher after the relaxation of face covering mandates. Other studies found no association between the implementation of facial covering policies and a reduction in COVID-19 cases [10, 19, 22, 30, 31]. Facial covering policies do not represent the actual use of masks for preventing infections but rather serve as public health measures. The impact of facial covering policies depends on compliance with the policies, proper mask usage, and the duration of mask-wearing. Bollyky et al. found no evidence that implementing facial covering policies reduced the number of COVID-19 infections, but they did observe an association between mask use and lower rates of COVID-19 infection [30]. Furthermore, it is important to note that a limitation of the mask evidence is the absence of standardized regulation or reporting regarding the type of masks employed. This variability in mask types may impact the assessment and comparison of mask impacts across studies.

The impact of school closures in reducing COVID-19 infections appears to be controversial. Liang et al. [22] and Li et al. [21] argued that school closures were associated with mitigating the spread of COVID-19, while Paireau et al. [11] and Ge et al. [19] suggested that their impact was limited. Conversely, Huy et al. [10] discovered that the policy of school closure had the opposite effect on the reduction of infection rate. A previous study found that the policy had a potential for effectively reducing influenza transmission [43]. However, the optimum strategy of the policy of school closures remains unclear, whether in controlling the spread of influenza or COVID-19 pandemic.

Liang et al. [22], Li et al. [21], and Ge et al [19]. considered workplace closure as an effective intervention in containing the spread of COVID-19, after the roll-out of coronavirus vaccines. Modeling studies estimated that implementing only workplace social distancing measures could reduce the median cumulative incidence of influenza in the general population by 23% from 2000 to 2017 [44].

Gathering restrictions are primarily implemented to curb the spread of infectious diseases by reducing interpersonal contact, which can occur through various transmission pathways such as droplets, direct contact, and aerosols [45]. A previous study indicated that restrictions on gathering had the greatest contribution (37.60%) to suppressing influenza transmission during the 2019–2020 influenza season [46]. Different levels of gathering restrictions have shown varying impact. According to the categorization by OxCGRT, the levels of restrictions on gatherings range from strictest to the weakest, including limitations on gatherings of 10 or fewer people, 11–100 people, 101–1000 people, and gatherings with 1000 or more individuals [1]. Studies by Huy et al. suggested that limiting gatherings to 10 or fewer people was most strongly correlated with a decrease in COVID-19 case numbers [10]. A similar finding was also supported by research conducted by Liang et al [22].

Stay-at-home orders [21], restrictions on international travel [19], public transport closures [10], vaccine mandates [30], and curfews [11] have been identified as effective measures in controlling the spread of COVID-19 according to a minority of included studies after the introduction of vaccines. Additionally, there is no evidence to suggest that restrictions on internal movement [10, 19, 21], public information campaigns [10, 22], and contact tracing [10, 22] were associated with a reduction in the transmission of COVID-19. Considering the number and heterogeneity of existing evidences, further research is needed to identify the impact and mechanisms of the implementation of these NPIs in controlling the spread of COVID-19.

There is controversy surrounding whether NPIs can effectively reduce COVID-19 deaths. Studies have shown that NPIs do not directly reduce the number of COVID-19 deaths [47]. However, research conducted by Hale et al. [20] and Wang et al. [25] found that, a higher stringency index was associated with a lower average daily death toll. It is possible that NPIs indirectly reduce the number of deaths by mitigating the spread of COVID-19. Nevertheless, these studies lack analysis or explanation regarding the specific indirect impacts.

The research on NPIs’ impact in reducing the transmission of infectious diseases, especially respiratory ones like SARS, influenza, and COVID-19, has always received attention. However, our understanding of the impact of these measures in controlling respiratory infectious diseases is still not comprehensive enough, even in the context of the ongoing COVID-19 pandemic, particularly since the introduction of vaccines.

### Strengths and limitations

This study has several strengths. Firstly, we conducted a systematic and comprehensive search across various databases to investigate the real-world impact of NPIs in containing the COVID-19 pandemic post-vaccine roll-out. Secondly, we employed a risk of bias assessment tool to critically assess the potential biases in the included studies. Thirdly, we summarized and analyzed the available evidence using quantitative and qualitative approaches, presenting the findings in tables and figures. Nonetheless, our study also has limitations. Firstly, we included three preprints that had not been peer-reviewed, although we did evaluate their risk of bias. Secondly, due to variations in study design, analytical methodologies, and outcome measures, we were unable to perform a meta-analysis and provide numerical estimates of impact. Thirdly, these studies primarily focused on the impact of NPIs during the first half of 2021. The available evidence be limited for conducting comparative analyses of the impact of NPIs at different stages of the epidemic curves or in communities utilizing different types of vaccines. Additionally, studies that evaluated the impact of NPIs included the post-vaccine rollout period but without considered vaccine coverage were excluded, despite the studies may provide insightful evidences. However, it is difficult to draw inferences about the impacts of NPIs after the vaccine rollout from these studies. Lastly, the evidence derived from the included studies was limited as they relied on retrospective and observational data, which cannot establish a causal relationship between NPIs and outcomes due to potential confounding variables.

## Conclusion

In conclusion, the understanding of the impact of NPIs in mitigating the pandemic post-vaccination is inadequate. NPIs had a larger contribution to the control of the pandemic as compared to vaccination during the early stage of vaccine implementation and in the context of the omicron variant. It is recommended to tailor NPIs based on factors like vaccination rates and variants with strong immune evasion, instead of lifting them suddenly, during early phases of vaccine roll-out in future pandemics. Various studies showed NPIs had varying impacts on curbing the COVID-19 pandemic. Policy- and decision-makers need to focus on the impact of different NPIs in diverse contexts, to determine when to ease or reinforce restrictions. It is essential to comprehend the policy mechanisms of these intervention measures in controlling the spread of COVID-19 and other respiratory infectious diseases, such as influenza.

### Electronic supplementary material

Below is the link to the electronic supplementary material.

Supplementary Material 1

## Data Availability

All data were collected from publicly available literatures, and all data generated or analyzed during this study are included in this article and its supplemental files.

## Abbreviations

NPIs: Non-pharmaceutical interventions
COVID-19: Coronavirus disease 2019
Rt: Time varying reproduction number

## References

[1] Hale T, Angrist N, Goldszmidt R, Kira B, Petherick A, Phillips T (2021). A global panel database of pandemic policies (Oxford COVID-19 Government Response Tracker). Nat Hum Behav.

[2] Mathieu E, Ritchie H, Ortiz-Ospina E, Roser M, Hasell J, Appel C (2021). A global database of COVID-19 vaccinations. Nat Hum Behav.

[3] Hsiang S, Allen D, Annan-Phan S, Bell K, Bolliger I, Chong T (2020). The effect of large-scale anti-contagion policies on the COVID-19 pandemic. Nature.

[4] Islam N, Sharp SJ, Chowell G, Shabnam S, Kawachi I, Lacey B et al. Physical distancing interventions and incidence of coronavirus disease 2019: natural experiment in 149 countries. BMJ. 2020;370. 10.1136/bmj.m2743 PMC7360923 32669358

[5] Liu Y, Morgenstern C, Kelly J, Lowe R, Jit M (2021). The impact of non-pharmaceutical interventions on SARS-CoV-2 transmission across 130 countries and territories. BMC Med.

[6] Bootsma MC, Ferguson NM. The effect of public health measures on the 1918 influenza pandemic in US cities. Proceedings of the National Academy of Sciences. 2007;104(18):7588-93. 10.1073/pnas.0611071104 PMC1849868 17416677

[7] Mendez-Brito A, El Bcheraoui C, Pozo-Martin F (2021). Systematic review of empirical studies comparing the effectiveness of non-pharmaceutical interventions against COVID-19. J Infect.

[8] Perra N (2021). Non-pharmaceutical interventions during the COVID-19 pandemic: a review. Phys Rep.

[9] Verelst F, Willem L, Beutels P (2016). Behavioural change models for infectious disease transmission: a systematic review (2010–2015). J Royal Soc Interface.

[10] Huy LD, Nguyen NTH, Phuc PT, Huang C-C (2022). The effects of non-pharmaceutical interventions on COVID-19 epidemic growth rate during pre-and post-vaccination period in Asian countries. Int J Environ Res Public Health.

[11] Paireau J, Charpignon M-L, Larrieu S, Calba C, Hozé N, Boëlle P-Y (2023). Impact of non-pharmaceutical interventions, weather, vaccination, and variants on COVID-19 transmission across departments in France. BMC Infect Dis.

[12] Tian D, Sun Y, Zhou J, Ye Q (2021). The global epidemic of the SARS-CoV-2 delta variant, key spike mutations and immune escape. Front Immunol.

[13] Shrestha LB, Foster C, Rawlinson W, Tedla N, Bull RA (2022). Evolution of the SARS-CoV‐2 omicron variants BA. 1 to BA. 5: implications for immune escape and transmission. Rev Med Virol.

[14] Zhang Y, Quigley A, Wang Q, MacIntyre CR. Non-pharmaceutical interventions during the roll out of covid-19 vaccines. BMJ. 2021;375. 10.1136/bmj.n2314 PMC8634371 34853011

[15] Moher D, Shamseer L, Clarke M, Ghersi D, Liberati A, Petticrew M (2015). Preferred reporting items for systematic review and meta-analysis protocols (PRISMA-P) 2015 statement. Syst Reviews.

[16] Dufault B, Klar N (2011). The quality of modern cross-sectional ecologic studies: a bibliometric review. Am J Epidemiol.

[17] Betran AP, Torloni MR, Zhang J, Ye J, Mikolajczyk R, Deneux-Tharaux C (2015). What is the optimal rate of caesarean section at population level? A systematic review of ecologic studies. Reproductive Health.

[18] Ford N, Holmer HK, Chou R, Villeneuve PJ, Baller A, Van Kerkhove M et al. Mask use in community settings in the context of COVID-19: a systematic review of ecological data. EClinicalMedicine. 2021;38. 10.1016/j.eclinm.2021.101024 PMC8287197 34308320

[19] Ge Y, Zhang W, Liu H, Ruktanonchai CW, Hu M, Wu X et al. Effects of worldwide interventions and vaccination on COVID-19 between waves and countries. Preprint. 2021.

[20] Hale T, Angrist N, Hale AJ, Kira B, Majumdar S, Petherick A (2021). Government responses and COVID-19 deaths: global evidence across multiple pandemic waves. PLoS ONE.

[21] Li H, Wang L, Zhang M, Lu Y, Wang W (2022). Effects of vaccination and non-pharmaceutical interventions and their lag times on the COVID-19 pandemic: comparison of eight countries. PLoS Negl Trop Dis.

[22] Liang L-L, Kao C-T, Ho HJ, Wu C-Y. COVID-19 case doubling time associated with non-pharmaceutical interventions and vaccination: a global experience. J Global Health. 2021;11. 10.7189/jogh.11.05021 PMC8442574 34552726

[23] Nesteruk I. Vaccination and testing as a means of ending the COVID-19 pandemic: comparative and statistical analysis. MedRxiv. 2022:2022.06. 16.22276531.

[24] Nesteruk I, Rodionov O (2022). Omicron waves of the COVID-19 pandemic and Effi Cacy of vaccinations and Testing. J ISSN.

[25] Wang C, Li H (2023). Variation in global policy responses to COVID-19: a bidirectional analysis. Int J Environ Res Public Health.

[26] Wang H, Lan Y (2023). The global dynamic transmissibility of COVID-19 and its influencing factors: an analysis of control measures from 176 countries. BMC Public Health.

[27] Ge Y, Zhang W-B, Wu X, Ruktanonchai CW, Liu H, Wang J (2022). Untangling the changing impact of non-pharmaceutical interventions and vaccination on European COVID-19 trajectories. Nat Commun.

[28] Kim S, Oh J, Tak S (2023). Association between face covering policies and the incidence of coronavirus disease 2019 in European countries. Osong Public Health Res Perspect.

[29] Zhou F, Hu T-J, Zhang X-Y, Lai K, Chen J-H, Zhou X-H (2022). The association of intensity and duration of non-pharmacological interventions and implementation of vaccination with COVID-19 infection, death, and excess mortality: natural experiment in 22 European countries. J Infect Public Health.

[30] Bollyky TJ, Castro E, Aravkin AY, Bhangdia K, Dalos J, Hulland EN (2023). Assessing COVID-19 pandemic policies and behaviours and their economic and educational trade-offs across US states from Jan 1, 2020, to July 31, 2022: an observational analysis. Lancet.

[31] Ertem Z, Nelson RE, Schechter-Perkins EM, Al-Amery A, Zhang X, Branch-Elliman W. Condition-Dependent and dynamic impacts of indoor masking policies for COVID-19 mitigation: a Nationwide, interrupted Time-Series Analysis. Clin Infect Dis. 2023:ciad115. 10.1093/cid/ciad115 37072937

[32] Shin J, Khuong QL, Abbas K, Oh J (2022). Impact assessment of mobility restrictions, testing, and vaccination on the COVID-19 pandemic in India. medRxiv.

[33] Kim K, Kim S, Lee D, Park C-Y (2023). Impacts of social distancing policy and vaccination during the COVID-19 pandemic in the Republic of Korea. J Economic Dynamics Control.

[34] He X, Liu H, Zeng F, Gao W. Factors influencing the trajectory of COVID-19 evolution: a longitudinal study of 12 Asian countries. medRxiv. 2023:2023.10. 20.23297319.

[35] Doroshenko A (2021). The combined effect of vaccination and nonpharmaceutical public health interventions—ending the COVID-19 pandemic. JAMA Netw Open.

[36] Alessandro C, Ferrone L, Squarcina M, Are. COVID-19 Containment Measures Equally Effective in Different World Regions? DISEI: Università degli Studi di Firenze; 2020.

[37] Wang X, Kong D, Guo M, Wang L, Gu C, Dai C (2021). Rapid SARS-CoV-2 nucleic acid testing and pooled assay by tetrahedral DNA nanostructure transistor. Nano Lett.

[38] Shao Z, Ma L, Bai Y, Tan Q, Liu XF, Liu S et al. Impact of mass rapid antigen testing for SARS-CoV-2 to mitigate Omicron outbreaks in China. J Travel Med. 2022;29(8). 10.1093/jtm/taac110 PMC9619432 36263876

[39] Embrett M, Sim SM, Caldwell HA, Boulos L, Yu Z, Agarwal G (2022). Barriers to and strategies to address COVID-19 testing hesitancy: a rapid scoping review. BMC Public Health.

[40] Lin L, Song Y, Wang Q, Pu J, Sun FY, Zhang Y (2021). Public attitudes and factors of COVID-19 testing hesitancy in the United Kingdom and China: comparative infodemiology study. JMİR Infodemiology.

[41] Song S, Zang S, Gong L, Xu C, Lin L, Francis MR (2022). Willingness and uptake of the COVID-19 testing and vaccination in urban China during the low-risk period: a cross-sectional study. BMC Public Health.

[42] Xin M, Lau JT-f, Lau MM (2022). Multi-dimensional factors related to participation in a population-wide mass COVID-19 testing program among Hong Kong adults: a population-based randomized survey. Soc Sci Med.

[43] Jackson C, Vynnycky E, Hawker J, Olowokure B, Mangtani P (2013). School closures and influenza: systematic review of epidemiological studies. BMJ open.

[44] Ahmed F, Zviedrite N, Uzicanin A (2018). Effectiveness of workplace social distancing measures in reducing influenza transmission: a systematic review. BMC Public Health.

[45] Wang CC, Prather KA, Sznitman J, Jimenez JL, Lakdawala SS, Tufekci Z (2021). Airborne transmission of respiratory viruses. Science.

[46] Ishola DA, Phin N (2011). Could influenza transmission be reduced by restricting mass gatherings? Towards an evidence-based policy framework. J Epidemiol Global Health.

[47] Mader S, Rüttenauer T (2022). The effects of non-pharmaceutical interventions on COVID-19 mortality: a generalized synthetic control approach across 169 countries. Front Public Health.

